# P-2106. A Pediatric Infectious Diseases Asynchronous eConsult Program: An Evaluation of Content, Impact Assessment and User Feedback

**DOI:** 10.1093/ofid/ofaf695.2270

**Published:** 2026-01-11

**Authors:** Vandana Madhavan, Wanlu Xu, Ann Marie Murray, Chadi El Saleeby

**Affiliations:** Massachusetts General Hospital, Boston, MA; Massachusetts General Hospital, Boston, MA; Massachusetts General Hospital, Boston, MA; Massachusetts General Hospital, Boston, MA

## Abstract

**Background:**

Asynchronous electronic consults, or eConsults, are a novel way for pediatric infectious disease (PID) physicians to provide non-urgent specialty advice to other clinicians. We sought to evaluate the first five years of a PID eConsult program with a specific focus on the utility, acceptability, and sustainability of the program.

**Methods:**

Data were abstracted from the electronic medical records for eConsults completed from March 2018 through October 2023 including patient and referring provider characteristics and consultation information. Recommendations were analyzed for content. Referring providers were surveyed to assess their experience and satisfaction with the eConsult service.

**Results:**

In the first five years of the program, 727 eConsults were completed. In 461 consultations (64.8%), the PID clinician suggested a change to the plan (new or additional diagnosis, diagnostic study, and/or therapeutic intervention) with 81.8% including counseling for the referring provider or patient. Of referring provider survey responders, 98.3% were satisfied/very satisfied with the eConsult program. Only 3.4% of respondents were dissatisfied with the turnaround time although 22% still found traditional “curbside” telephone calls to be more valuable than eConsults. Both the number of individual referring providers and the number of referring practices increased steadily over five years.

**Conclusion:**

Asynchronous eConsults present a novel platform for PIDspecialists to formally provide recommendations. A majority of recommendations changed the diagnostic plan and treatment while providing education to both patients and referring providers. Our program demonstrated acceptance of this consulting format from referring providers and sustainability over the first five years of the program.

**Disclosures:**

All Authors: No reported disclosures

